# Aberrant Expression of Xist in Aborted Porcine Fetuses Derived from Somatic Cell Nuclear Transfer Embryos

**DOI:** 10.3390/ijms151221631

**Published:** 2014-11-25

**Authors:** Lin Yuan, Anfeng Wang, Chaogang Yao, Yongye Huang, Feifei Duan, Qinyan Lv, Dongxu Wang, Hongsheng Ouyang, Zhanjun Li, Liangxue Lai

**Affiliations:** 1 Jilin Provincial Key Laboratory of Animal Embryo Engineering, College of Animal Sciences, Jilin University, Changchun 130062, China; E-Mails: peguin119@126.com (L.Y.); walfers@163.com (A.W.); lengyinfei@126.com (C.Y.); huangyongye88@163.com(Y.H.); dff1125@gmail.com (F.D.); lvqingyan123@163.com (Q.L.); wang_dong_xu@163.com (D.W.); ouyh@jlu.edu.cn (H.O.); 2 Key Laboratory of Regenerative Biology, South China Institute for Stem Cell Biology and Regenerative Medicine, Guangzhou Institutes of Biomedicine and Health, Chinese Academy of Sciences, Guangzhou 510530, China

**Keywords:** porcine, somatic cell nuclear transfer, DNA methylation, Xist

## Abstract

Cloned pigs generated by somatic cell nuclear transfer (SCNT) show a greater ratio of early abortion during mid-gestation than normal controls. X-linked genes have been demonstrated to be important for the development of cloned embryos. To determine the relationship between the expression of X-linked genes and abortion of cloned porcine fetuses, the expression of X-linked genes were investigated by quantitative real-time polymerase chain reaction (q-PCR) and the methylation status of Xist DMR was performed by bisulfate-specific PCR (BSP). q-PCR analysis indicated that there was aberrant expression of X-linked genes, especially the upregulated expression of Xist in both female and male aborted fetuses compared to control fetuses. Results of BSP suggested that hypomethylation of Xist occurred in aborted fetuses, whether male or female. These results suggest that the abnormal expression of Xist may be associated with the abortion of fetuses derived from somatic cell nuclear transfer embryos.

## 1. Introduction

Since the first cloned piglets were generated in 2000, a large number of genetically modified SCNT cloned pigs have been harvested in a number of laboratories worldwide [1,2,3]. SCNT is a useful technique for embryos production in many fields, such as animal models, xenotransplantation and embryonic stem cell research. Although many genetically modified cloned pigs have been generated, there are still problems that must be resolved. At present, the general cloning efficiency remains unsatisfactory, at lower than 1.5%, although it is improving [4]. The majority of SCNT embryos are lost during pregnancy, including embryos dying before implantation and fetal resorption. A certain proportion of surrogate sows for transferred SCNT embryos suffer abortions during gestation, and these may be concentrated between Day 27 and Day 34 following SCNT [5]. Additionally, some cloned piglets suffer from serious developmental abnormalities and die within a few days of birth.

It is well known that proper gene expression is important for pregnancy maintenance and fetal survival. Because the somatic nucleus does not pass through the germ-cell developmental program in which epigenetic modifications are normally reset [6], the epigenetic modifications may not be fully reestablished in some SCNT cloned embryos. The low efficiency of porcine SCNT seems to result largely from incomplete reprogramming of the donor cell nucleus transferred into the recipient oocyte. In particular, X-linked genes were susceptible to epigenetic errors during the SCNT process in many studies [7]. A previous report revealed that aberrant expression patterns of X inactivation-specific transcript (Xist), glucose-6-phosphate dehydrogenase (G6PD), hypoxanthine guanine phosphoribosyltransferase (HPRT1) and V-raf murine sarcoma 3,611 viral oncogene homolog 1 (ARAF1) were prevalent in the deceased neonatal piglets [8]. Among these genes, Xist is considered to be one of the first imprinted genes that expressed during zygotic genome activation [9], and it is aberrantly expressed in cloned mouse embryos [10,11]. Importantly, it was reported that the cloning efficiency could be significantly improved by regulating the expression of the Xist transcript [12,13]. G6PD is a housekeeping X-linked gene whose main function is to produce NADPH, which is involved in the normal processing of carbohydrates; HPRT is also an X-chromosome-linked enzyme, which plays a central role in metabolism [14]. On the human X chromosome, HPRT is located more closely to the (XIC) than G6PD [15]. ARAF1 is involved in cell growth and development [16]. It is located on porcine chromosome Xp13 [17]. All these reports showed an important role of Xist in pre-implantation embryonic development.

In the present study, for better understanding the early abortion and improving cloning efficiency of porcine SCNT, we determined the relationship between the expression of X-linked genes and abortion of cloned porcine fetuses, the expression of X-linked genes and the methylation patterns of Xist DMR were examined in the porcine abortion samples.

## 2. Results and Discussion

### 2.1. Results

#### 2.1.1. Sample Collection and Sex Identification of Aborted Fetuses

**Figure 1:**
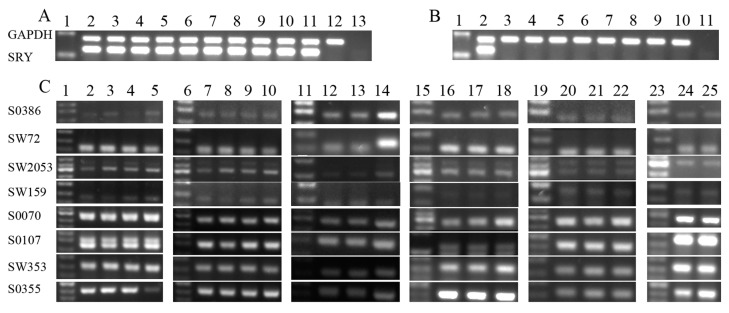
Genotypic identification of aborted fetuses by PCR analysis. (**A**) Glyceraldehyde-3-phosphate dehydrogenase (GAPDH) was included as a loading control (upper ladder). SRY gene amplification by PCR (lower ladder). Lane 1: 2000-bp ladder as DNA ladder. Group 1: donor cell, lane 2; samples, lanes 3, 4 and 5. Group 2: donor cell, lane 6; samples, lanes 7, 8 and 9. Group 3: donor cell, lane 10; samples, lanes 11 and 12. Lane 13: amplification of ddH_2_O; (**B**) GAPDH was included as a loading control (upper ladder). SRY gene amplification by PCR (lower ladder). Lane 1: 2000-bp ladder as a DNA size marker. Lane 2: donor cell from group 1. Group 4: donor cell, lane 3; samples, lanes 4 and 5. Group 5: donor cell, lane 6; samples, lanes 7 and 8. Group 6: donor cell, lane 9; sample, lane 10. Lane 11: amplification of ddH_2_O and (**C**) Microsatellite marker analysis was used to identify the genotype of the aborted fetuses and corresponding donor cells for somatic cell nuclear transfer. Lanes 1, 6, 11, 15, 19 and 23: 50-bp ladder as a DNA size marker. Group 1: donor cell, lane 2; samples, lanes 3, 4 and 5. Group 2: donor cell, lane 7; samples, lanes 8, 9 and 10. Group 3: donor cell, lane 12; samples, lanes 13 and 14. Lane 12 was donor cell of lanes 13 and 14 (group 3). Group 4: donor cell, lane 16; samples, lanes 17 and 18. Group 5: donor cell, lane 20; samples, lanes 21 and 22. Group 6: donor cell, lane 24; sample, lane 25.

Considering that there was possibly gene expression discrepancy between male and female aborted fetus, sex identification was performed with genomic DNA from the aborted fetuses and SCNT donor cells. As shown in Figure 1A,B, the gender of the mostly aborted fetuses were consistent with their donor cells, however, the SRY gene failed to be amplified in one aborted fetus derived from the male donor cell, possibly indicating that some of these samples were from parthenogenetic fetuses. To verify this hypothesis, microsatellite loci in aborted fetuses and donor cells were analyzed. The results of the microsatellite analysis revealed that one of the 13 aborted fetuses was from a parthenogenetic embryo (Figure 1C), suggesting that the incomplete enucleation may have occurred during the physical enucleation step of the SCNT process. In the following study, the parthenogenetic sample was excluded.

#### 2.1.2. Transcription Patterns of X-Linked Genes in Aborted Fetuses

**Figure 2:**
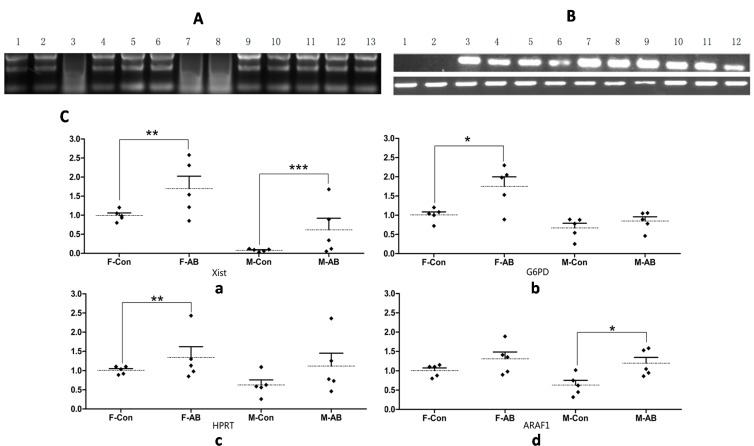
Transcription patterns of X-linked genes. (**A**) The electrophoresis pattern of total RNA extracted from the samples. Lanes 1–3, 4–6, 7–8, 9–10, 11–12 and lane13 represented group 1, 2, 3, 4, 5 and 6 respectively. Three bands represented the high quality of RNA, the smear band showed the degraded RNA; (**B**) Xist gene amplification by RT-PCR (upper ladder). GAPDH was included as an internal control (lower ladder). Lanes 1: male fetus derived from normal fertilization; Lanes 2–6: male aborted fetuses; Lanes 7: female fetuses derived from normal fertilization; Lanes 8–12: female aborted fetuses and (**C**) Gene expression levels of (**a**) X inactivation-specific transcript (Xist); (**b**) glucose-6-phosphate dehydrogenase (G6PD); (**c**) hypoxanthine guanine phosphoribosyltransferase (HPRT1) and (**d**) V-raf murine sarcoma 3611 viral oncogene homolog 1(ARAF1) were determined by q-PCR. * denotes *p* < 0.05; ** denotes *p* < 0.01; *** denotes *p* < 0.001. Dotted line: the average level of gene expression. Solid line: standard deviation.

In the present study, 13 aborted fetus samples were collected (Table 1). As the analyzed samples were aborted porcine fetuses, the integrity of RNA was proved firstly by 1% agarose gel electrophoresis. As shown in Figure 2A, the RNA in line 3, 7 and 8 were degrade, so here we used the sample of 1, 2, 4, 5, 6, 9, 10, 11, 12 and 13 for the following study (Figure 2A). To determine the expression levels of X-linked genes in aborted fetuses, the expression of four X-linked gene transcripts were assessed by q-PCR. As shown in Figure 2C, there was no significant difference expression of G6PD, HPRT1 and ARAF1 between control female and male fetuses, while the expression of Xist was almost loss in control male fetuses. However, the transcripts of Xist, G6PD, HPRT1 and ARAF1 showed a different tendency between control and aborted samples. In particular, the expression of Xist mRNA was obviously higher in both female and male aborted fetuses than those of control fetuses. To confirm this result, the expression of Xist was also detected by RT-PCR (Figure 2B). The RT-PCR results revealed that the expression of Xist was barely detectable in control male fetuses and in one male aborted fetus, but Xist expression could be detected in both control and aborted female fetuses. The expression of G6PD was 1.8-fold higher in aborted than control female fetuses, while there was no significant difference between male control and aborted fetuses. The expression status of HPRT1 was similar to G6PD. However, the expression of ARAF1 was higher in male aborted fetuses than in male control fetuses, and there was no significant difference between female control and aborted fetuses (Figure 2C). These results indicate that proper regulation of the expression of Xist may be critical for the development of cloned fetuses.

**Table 1:** Collection and analysis of the aborted porcine samples.

Groups	No. of Samples	Gender of the Donor Cell	SRY Amplification *	q-PCR ^&^
1	3	Male	3	2
2	3	Male	3	3
3	2	Male	1	0
4	2	Female	0	2
5	2	Female	0	2
6	1	Female	0	1

* The number of samples in which the SRY gene could be detected; ^&^ The number of samples used in the q-PCR examination.

#### 2.1.3. Methylation Status of the Xist Gene in Male and Female Aborted Fetuses

**Figure 3:**
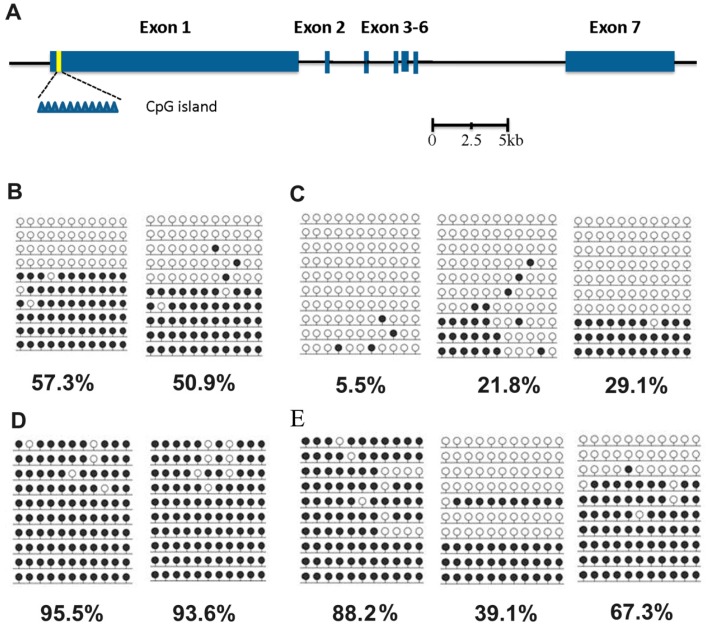
Methylation status of Xist DMR. (**A**) Gene structure and precise location of the CpGs in the porcine Xist gene. Blue rectangles represent the seven exons of porcine Xist, the yellow region represents the location of CpG island. The eleven blue triangles represent the number of CpG within the DMR; (**B**) Control female fetuses; (**C**) Aborted female fetuses; (**D**) Control male fetuses and (**E**) Male aborted fetuses. A filled black circle indicates a methylated CpG dinucleotide, open circle indicates an unmethylated CpG dinucleotide.

The q-PCR results indicate that the Xist was significantly over-expressed in aborted samples. To confirm this data, the methylation status of Xist DMR was determined by BSP. A model of gene structure and precise location of the CpGs analyzed was proposed firstly in this study using the GenBank and Ensembl databases (Figure 3A). BSP results indicated that full methylation status of Xist DMR in male control fetuses (Figure 3B) while partial methylation in female aborted fetuses (Figure 3C). Compared to the controls, the hypermethylation status of Xist DMR was also observed in both aborted female and male fetuses, which is consisted with the q-PCR data in Figure 2B. Based on these results, it seemed that the abnormal methylation of Xist DMR may account for the abortion of SCNT fetuses.

### 2.2. Discussion

It is commonly believed that the epigenetic errors of donor genome are the major problem underlying low SCNT efficiency and the abortion of cloned fetuses [18,19,20]. Early embryos vary in their responses to different environments, and fetuses with defects may suffer abortion more easily. Actually, in cat, a certain number of proteins are abnormally expressed in 21-day-old placentas of fetuses derived from cloned embryo transfer, and this phenomenon is suggested to be associated with fetal loss during pregnancy [21]. Recent studies have suggested that most X-linked genes may function not only in sex-related transcriptional differences, but also in the regulation of autosomal gene expression in fetuses. The expression of X-linked genes is abnormal in porcine IVF and cloned embryos, suggesting that the regulation of X-linked genes is susceptible to *in vitro* culture and the SCNT process, thus leading to aberrant subsequent embryonic development [7]. Therefore, the expression of X-linked genes likely plays a critical role in embryonic development. In the present study, the samples derived from aborted porcine SCNT cloned embryos were collected and subjected to q-PCR and BSP analysis.

The q-PCR amplification data indicated that four X-linked genes, Xist, G6PD, HPRT1 and ARAF1, showed different expression patterns in female and/or male aborted fetuses. These apparent discrepancies may indicate that abnormal expression of these genes was associated with abortion. In our study, the upregualted expression of Xist was detected in both male and female aborted fetues. Xist is known to play a crucial role in X-chromosome inactivation (XCI). Studies have revealed that the XCI occurs in embryo development, which can be regulated by Xist during the course of reprogramming [22,23]. Recently, many studies have highlighted the importance of Xist RNA for early embryo development. It was found that the Xist and X-linked genes were abnormally expressed in a cloned mouse [12], and down-regulation of Xist during early embryo development could greatly enhance the birth rates in cloned mice [12,13]. It is possible that the aberrant expression of Xist was one of the crucial factors contributing to the abortion of fetuses derived from SCNT cloned embryos.

The expression status of HPRT1 was similar to that of G6PD, perhaps in part because they are close together on the chromosome and both playing important roles in metabolism. Consistent with our results, the expression levels of G6PD and HPRT1 were shown to be higher in bovine female morulae and blastocysts than those in males [14]. In contrast, the expression level of ARAF1 was higher in male aborted fetuses than in male control fetuses. However, it has been demonstrated that knockdowns Xist is a global effect, not only on the X-chromosome but also autosomal expression in cloned mouse embryos, which is consistent with previous findings [12]. In our study, despite the upregulated expression of Xist was observed in aborted cloned embryos, we could not find repressed patterns of the X-linked genes. Although conflicting results exist, the expression relationship between Xist and X-linked genes a more complicated and diversification. It is reported Xist RNA-independent initiation of X-chromosome inactivation [24] and over 15% of X-linked genes “escape” from XCI and continue to be expressed from the inactive X chromosome in human [25]. In addition, there is no direct regulation relationship between Xist and the X-linked genes in SCNT embryos [12,26]. So here we deduce that the fault reprogramming of cloned embryos could possibly exacerbate the “escape” from XCI, and much more effort should be exerted on XCI so as to improve the development of SCNT embryos.

The unfaithful maintenance of gene expression and DNA methylation in cloned fetuses is indicated as a result of aberrant reprogramming during SCNT [27]. DNA methylation is crucial for embryo development and function in the transcriptional inactivation of certain genes and transposons [28,29]. In this study, the BSP result demonstrated the methylation levels of Xist were lower in aborted fetuses than in controls, which indicted changes of methylation status may account for abortions of cloned porcine fetuses. It should be noted that one of the male aborted fetuses also showed hypermethylation status (Figure 3E), which is consistent with the results of Xist was expressed at a similar level between aborted and control fetuses (Figure 2B), indicating that other potential factors likely also lead to the abortion of cloned fetuses. Taken together, in addition to DNA methylation, histone acetylation, phosphorylation and ubiquintination may also be abnormal in aborted fetuses, and these epigenetic modifications should be studied in further research.

## 3. Experimental Section

### 3.1. Ethics Statement

The pig experiments were carried out in accordance with the guidelines on animal care and use of animals and were supervised by the Animal Care and Use Committee of Jilin University, with approval number 2012-009.

### 3.2. Samples Collection

Fetuses were collected at 33 to 35 days from the mated sow after fertilization. Fetuses were cut into small pieces after discarding the head, viscera, limbs and tail, and were then digested with DNase I (25 kU/mL) and collagenase IV (200 IU/mL). When confluence was reached, cells were frozen in medium composed of 90% fetal bovine serum (FBS) and 10% dimethyl sulfoxide (DMSO; Amresco, Solon, OH, USA); the cells were stored in liquid nitrogen for the donor cell of SCNT.

The porcine ovaries were collected from a local slaughterhouse in Changchun city, transported to the laboratory at 32–37 °C in physiological saline solution within 2–3 h. The cumulus-oocyte complexes (COCs) were obtained using an 18-gauge needle attached to a 10-cc syringe. The COCs possessing three to five layers of cumulus cells were selected and washed three times in TL-HEPES-PVA solution. After maturation for 42–44 h, oocytes with the first polar body were selected for SCNT recipients [30].

The selected MII oocytes were transferred to manipulation medium supplemented with 7.5 mg/mL cytochalasin B, and covered with mineral oil. Enucleation was accomplished by aspirating the polar body and MII chromosomes with a small amount of the surrounding cytoplasm. A single donor cell showing normal morphology was then transferred into the perivitelline space and gently placed adjacent to the recipient cytoplasm. For fusion and activation, the cell-cytoplast complexes were pooled in a chamber filled with fusion medium, and 2 DC pulses of 1.2 kV/cm for 30 µs were then applied through a BTX Electro Cell Manipulator 2001 (BTX, San Diego, CA, USA). Approximately 50 reconstructed embryos were transferred into 100 µL PZM3 and covered with mineral oil, then cultured at 39 °C with 5% CO_2_.

After being cultured in PZM3 at 39 °C for 16–18 h, the reconstructed embryos were transferred to the oviducts of surrogate sows on the day of the onset of estrus. Ultrasound examination was performed to verify the pregnancy of the sows after embryo transfer. The surrogate sows were maintained carefully and observed twice daily. The natural occurrence of abortion was confirmed through observation, and the aborted sample was collected immediately. The aborted sample was immediately transported to the laboratory. After rinsing with sterile water, the aborted fetuses were used to total RNA and/or genomic DNA extraction. Fetuses derived from normal fertilized sows were used as the controls.

### 3.3. Genotype Identification and Bisulfate-Specific PCR (BSP)

The genomic DNA was isolated using TIANmp Genomic DNA Kit (Tiangen, Beijing, China) following the manufacturer’s instruction.

Sex identification of the fetuses was performed by amplifying the male determining gene SRY. The reaction was performed as follows: pre-denaturation at 95 °C for 3 min followed by 30 cycles of denaturation at 95 °C for 30 s, annealing at 58 °C for 30 s, and extension at 72 °C for 30 s. The GAPDH was used as a control to verify the quality of DNA extraction. The primers are shown in Table 2.

Microsatellites were used to identify the inheritance patterns. Microsatellite analysis was performed for genomic DNA obtained from aborted fetuses and for the SCNT donor cells. Eight microsatellite loci (S0386, SW72, SW2053, SW353, SW159, S0070, S0107, and S0355) located on different porcine chromosomes were amplified, and then analyzed by 3% agarose gel electrophoresis. These primers are shown in Table 3.

**Table 2:** Primer sequences for reversetranscription PCR (RT-PCR).

Gene	Primer Sequence	Size (bp)
SRY	Sense: GCTTTCATTGTGTGGTCTCGT	309
	Antisense: CTTGGCGACTGTGTATGTGAAG	
GAPDH	Sense: GATGGCCCCTCTGGGAAACTGTG	404
	Antisense: GGACGCCTGCTTCACCACCTTCT	
Xist	Sense: GGATAATATGGTTGATTTTGTTATGTG	212
	Antisense: CCACCACCCTTTCTAATTAAATATATC	

**Table 3:** Primer sequences for microsatellites.

Locus	Primer Sequence
S0386	Sense: 5-GAACTCCTGGGTCTTATTTTCTA-3
	Antisense: 5-GTCAAAAATCTTTTTATCTCCAACAGTAT-3
SW72	Sense: 5-ATCAGAACAGTGCGCCGT-3
	Antisense: 5-TTTGAAAATGGGGTGTTTCC-3
SW2053	Sense: 5-AAGCAAGGTGCCACTGTTG-3
	Antisense: 5-CGAACCCGATGTCCTCTGAC-3
SW159	Sense: 5-GATTGGGAATTTGGGGTT-3
	Antisense: 5-CGTCTTTACTTTTGTTGTTACG-3
S0070	Sense: 5-GGCGAGCATTTCATTCACAG-3
	Antisense: 5-GAGCAAACAGCATCGTGAGC-3
S0107	Sense: 5-CAAGGATGCCTGTAACTGGTGCAG-3
	Antisense: 5-TCCTTAAGGCCTCGTAGGATCTGT-5
SW353	Sense: 5-CACCCCATGCCTGAATACTG-3
	Antisense: 5-ATGTGAAGACTCATGCTTGGG-3
S0355	Sense: 5-TCTGGCTCCTACACTCCTTCTTGATG-3
	Antisense: 5-GTTTGGGTGGGTGCTGAAAAATAGGA-3

BSP was performed to determine the methylation of Xist DMR. The CpGenomeTM Turbo Bisulfite Modification Kit (Millipore, Jaffrey, NH, USA) was used for bisulfate treatment according to the manufacturer’s instructions. The BSP primers of Xist DMR were shown in Table 4. The PCR products were purified using the TIANgel Midi Purification Kit (Tiangen, Beijing, China) and cloned into the PGM-T vector (Tiangen, Beijing, China). Ten positive plasmid clones were sequenced at Tiangen Corporation.

**Table 4:** Primer sequence for Bisulfate-Specific (BS)-PCR [31].

Gene	Primer Sequence	Size (bp)
Xist outside	Sense: GTGTGTATTTTTTGATAAATTTTGT	330
	Antisense: CTATACTAACTAACTAAATAAAAAC	
Xist inside	Sense: GGATAATATGGTTGATTTTGTTATGTG	212
	Antisense: CACCACCCTTTCTAATTAAATATATC	

### 3.4. Gene Expression Analysis

Total RNA extraction was performed using TRNzol-A+ reagent (Tiangen, Beijing, China) according to the manufacturer’s instructions. The integrety of RNA was confirmed by 1% agarose gel electrophoresis. The residual DNA was digested with DNase I. cDNA synthesis was then performed with the BioRT cDNA First Strand Synthesis kit (Bioer Technology, Hangzhou, China).

RT-PCR amplification was performed with the following reaction conditions: Pre-denaturation at 94 °C for 3 min; followed by 30 cycles of denaturation at 94 °C for 30 s, annealing at 60 °C for 30 s, and extension at 72 °C for 30 s.

Quantitative real-time PCR (q-PCR) was performed using the BioEasy SYBR Green I Real Time PCR Kit (Bioer Technology, Hangzhou, China) with the BIO-RAD iQ5 Multicolor Real-Time PCR Detection System. Forty cycles of amplification were performed under the following reaction conditions: initial denaturation at 95 °C for 5 min; followed by 40 cycles of denaturation at 94 °C for 10 s, annealing at 55 °C for 15 s, and extension at 72 °C for 30 s. GAPDH was used as an endogenous control in this study. Primers for q-PCR are listed in Table 5. The 2^−∆∆*C*t^ method [32] was used to analyze the gene expression. For each gene examined, five samples were included, and the data were obtained from three replicate experiments.

**Table 5:** Primer sequence of Xist, G6PD, HPRT1 and ARAF1 for q-PCR [8].

Gene	Primer Sequence	Size (bp)
Xist	Sense: GAAGAGATGCTCCAGGCCAAT	87
	Antisense: AGGTGTTGCTGGCTGATGCT	
G6PD	Sense: CCTCCTGCAGATGCTGTGTCT	112
	Antisense: CGCCTGCACCTCTGAGATG	
HPRT1	Sense: CGTCTTGCTCGAGATGTGATG	98
	Antisense: TCCAGCAGGTCAGCAAAGAA	
ARAF1	Sense: CGGGATGGCATGAGTGTCTAC	108
	Antisense: GACTGTCTTTCGCCCCTTGA	
GAPDH	Sense: ATTCCACGGCACAGTCAAGG	120
	Antisense: ACATACTCAGCACCAGCATCG	

### 3.5. Statistical Analysis

All experiments were repeated at least three times. The gene expression between the aborted fetuses and normal controls were compared using Student’s *t*-test with SPSS 16.0 software (SPSS Inc., Chicago, IL, USA).

## 4. Conclusions

Upregulated expression of Xist and aberrant expression of X-linked genes were detected in both female and male aborted fetuses, suggesting that altered expression of X-linked genes were possibly accounted for the failure development of porcine SCNT embryos. It is quite common for the occurrence of epigenetic errors during SCNT process, and hypomethylation status of the Xist gene in aborted fetuses in this study. In all, incomplete epigenetic reprogramming of X-linked genes may affect the subsequent development of porcine SCNT embryos. To improve the development of porcine SCNT embryos, it is necessary to restore X-linked genes to the normal expression level.
